# Peripheral Blood Gene Expression as a Novel Genomic Biomarker in Complicated Sarcoidosis

**DOI:** 10.1371/journal.pone.0044818

**Published:** 2012-09-12

**Authors:** Tong Zhou, Wei Zhang, Nadera J. Sweiss, Edward S. Chen, David R. Moller, Kenneth S. Knox, Shwu-Fan Ma, Michael S. Wade, Imre Noth, Roberto F. Machado, Joe G. N. Garcia

**Affiliations:** 1 Institute for Personalized Respiratory Medicine, The University of Illinois at Chicago, Chicago, Illinois, United States of America; 2 Section of Pulmonary, Critical Care, Sleep and Allergy, Department of Medicine, The University of Illinois at Chicago, Chicago, Illinois, United States of America; 3 Institute of Human Genetics, The University of Illinois at Chicago, Chicago, Illinois, United States of America; 4 Department of Pediatrics, The University of Illinois at Chicago, Chicago, Illinois, United States of America; 5 Section of Rheumatology, Department of Medicine, The University of Illinois at Chicago, Chicago, Illinois, United States of America; 6 Division of Pulmonary and Critical Care Medicine, Department of Medicine, The Johns Hopkins University, Baltimore, Maryland, United States of America; 7 Section of Pulmonary and Critical Care, Department of Medicine, The University of Arizona, Tuscon, Arizona, United States of America; 8 Section of Pulmonary/Critical Care, Department of Medicine, The University of Chicago, Chicago, Illinois, United States of America; University of Giessen Lung Center, Germany

## Abstract

Sarcoidosis, a systemic granulomatous syndrome invariably affecting the lung, typically spontaneously remits but in ∼20% of cases progresses with severe lung dysfunction or cardiac and neurologic involvement (complicated sarcoidosis). Unfortunately, current biomarkers fail to distinguish patients with remitting (uncomplicated) sarcoidosis from other fibrotic lung disorders, and fail to identify individuals at risk for complicated sarcoidosis. We utilized genome-wide peripheral blood gene expression analysis to identify a 20-gene sarcoidosis biomarker signature distinguishing sarcoidosis (n = 39) from healthy controls (n = 35, 86% classification accuracy) and which served as a molecular signature for complicated sarcoidosis (n = 17). As aberrancies in T cell receptor (TCR) signaling, JAK-STAT (JS) signaling, and cytokine-cytokine receptor (CCR) signaling are implicated in sarcoidosis pathogenesis, a 31-gene signature comprised of T cell signaling pathway genes associated with sarcoidosis (TCR/JS/CCR) was compared to the unbiased 20-gene biomarker signature but proved inferior in prediction accuracy in distinguishing complicated from uncomplicated sarcoidosis. Additional validation strategies included significant association of single nucleotide polymorphisms (SNPs) in signature genes with sarcoidosis susceptibility and severity (unbiased signature genes - *CX3CR1*, *FKBP1A*, *NOG*, *RBM12B*, *SENS3*, *TSHZ2*; T cell/JAK-STAT pathway genes such as *AKT3*, *CBLB*, *DLG1*, *IFNG*, *IL2RA*, *IL7R*, *ITK*, *JUN*, *MALT1*, *NFATC2*, *PLCG1*, *SPRED1*). In summary, this validated peripheral blood molecular gene signature appears to be a valuable biomarker in identifying cases with sarcoidoisis and predicting risk for complicated sarcoidosis.

## Introduction

Individuals with sarcoidosis, a systemic inflammatory and non-caseating granulomatous disease of unknown origin affecting multiple organs and invariably the lung [1], [2], typically undergo spontaneous resolution. However, ∼20% of affected individuals experience progressive disease with respiratory, cardiac or nervous system involvement. Complicated sarcoidosis is defined as exhibiting either cardiac manifestations (e.g., ventricular arrhythmias) [3], neurologic involvement (e.g., with evidence of hyperdense MRI lesions) [4] or deteriorating lung function (e.g., FVC <50%). Currently, FDA-approved therapies for complicated sarcoidosis do not exist and corticosteroids and corticosteroid-sparing immunosuppressive agents (TNFα inhibitors) have met with only limited success [5]. The accurate identification of individuals with or at risk for complicated sarcoidosis is a vexing clinical challenge with attempts to define clinically-useful biomarkers largely unsuccessful. Sarcoidosis biomarkers are desperately needed to deliver targeted therapies in individuals with complicated sarcoidosis and to identify patients at risk for increased morbidity and significant mortality as a consequence of complicated sarcoidosis.

Previous sarcoidosis candidate gene studies focused on granuloma formation and immune response pathways implicated several genes linked to sarcoidosis susceptibility [6] including HLA antigens such as class I HLA-B8 [7] and HLA-DRB1 [6], [8], [9]. Additional candidate genes involved in antigen processing, antigen presentation, macrophage and T-cell activation, and injury repair have also been associated with sarcoidosis susceptibility [10]–[23]. Whole genome scanning studies based upon unbiased, genome-based approaches identified genes implicated in sarcoidosis susceptibility via linkage analysis (i.e., D6S1666 in 63 German families with affected siblings) [24] with further scanning suggesting rs2076530 in *BTNL2* (butyrophilin-like 2) gene to be associated with sarcoidosis development [25]. A significant challenge remains, however, in the assessment of sarcoidosis susceptibility in specific high-risk populations as well as in the identification of sarcoidosis patients at risk for complicated, progressive disease.

Our study was designed to identify novel genomic biomarkers by comparing genome-wide gene expression data in African American (AA) and European descent ancestry (EA) sarcoidosis cases. We identified a universal gene signature that differentiates sarcoidosis patients from healthy controls and distinguishes complicated sarcoidosis (pulmonary- FVC<50%, cardiac, or neurologic sarcoidosis) from uncomplicated sarcoidosis. This gene signature was superior in prediction accuracy in each of the AA and EA populations when compared to a second signature comprised of genes within the T cell receptor–innate immunity pathway that includes genes previously associated with sarcoidosis. These signatures distinguished sarcoidosis patients from idiopathic pulmonary fibrosis (IPF) cases with signature validation provided by significant association of genetic variants within signature genes with sarcoidosis susceptibility. These results highlight the utility of peripheral blood molecular gene signatures as valuable biomarkers for predicting individuals at risk for complicated sarcoidosis and for potentially facilitating individualized therapies in this enigmatic disorder.

## Results

### Patient Characteristics

PBMC samples were collected from subjects with sarcoidosis (n = 39) and healthy controls (n = 35) (Table 1). The clinical characteristics of study patients are displayed in Table 2. Significant differences in age, gender, race and pulmonary function studies did not exist between uncomplicated and complicated sarcoidosis cases (P>0.05 by χ2 test for gender and p>0.05 by t-test for the other characteristics). Uncomplicated sarcoidosis cases trended toward higher corticosteroid usage whereas complicated sarcoidosis cases trended toward higher methotrexate usage and were more likely to be receiving anti-TNFα therapy. However, these differences were not statistically significant (P>0.05 for all drugs) (Table 2). Predictably, complicated pulmonary sarcoidosis cases exhibited significantly reduced pulmonary function compared to the other study groups (data not shown).

**Table 1:** Study subjects with racial and complication status.

Population	Healthy controls	Uncomplicated cases	Complicated cases		
			Cardiac	Neurologic	FVC<50%
AA	8	11	5	5	10
EA	27	6	3	2	1
Total	35	17	8	7	11

Amongst the patients with cardiac sarcoidosis, three had severe pulmonary disease. In the patients with neurologic sarcoidosis, five had pulmonary disease. EA: European Americans (Caucasians); AA: African Americans.

**Table 2:** Patient characteristics and concomitant medications.

Characteristics	Uncomplicated sarcoidosis (n = 17)	Complicated sarcoidosis(n = 22)
Age	49±10	47±9
Gender (Male/Female)	5/12	5/17
FVC, L	2.9±0.8	2.7±1.5
FVC, percent of predicted	74±17	65±31
FEV_1_, L	2.2±0.7	2.1±1
FEV1, percent of predicted	74±17	67±30
DL_CO_, percent of predicted	74±23	65±28
Corticosteroids, n (dose, mg prednisone equivalent per day)	7 (20±16)	11 (13±11)
Methotrexate, n (dose, mg per week)	3 (12.25±3.5)	7 (11±4)
Mycophenolate, n (dose, mg per day)	1 (250)	3 (667±289)
Anti-TNF alpha therapy, n	0	3

### Identification of Differentially-expressed Genes in Sarcoidosis

**Figure 1:**
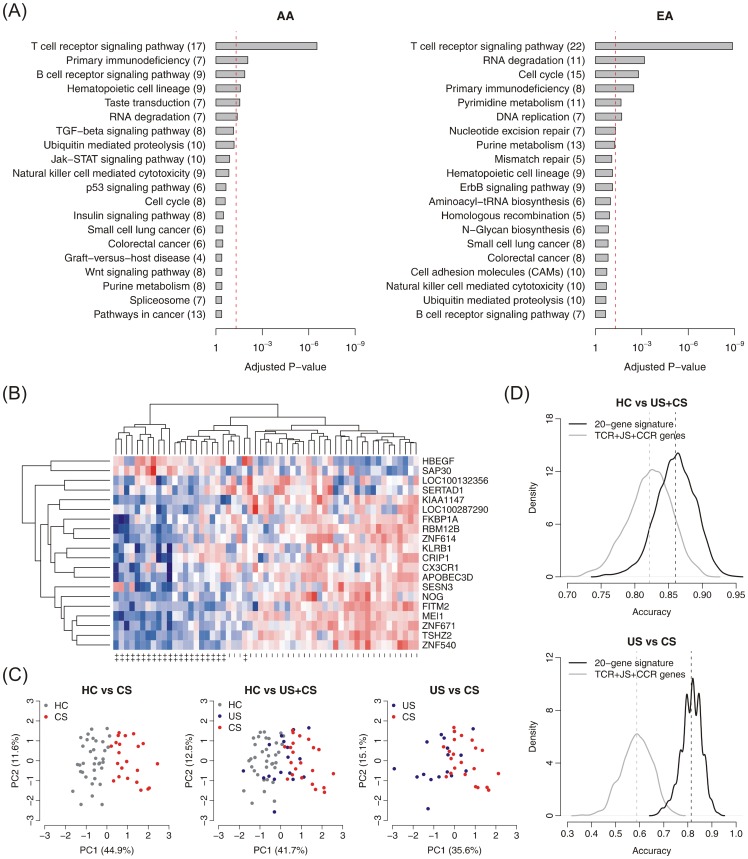
Identifying gene signatures in sarcoidosis. Panel A. Enriched pathways among complicated sarcoidosis-associated genes. The top ranking KEGG pathways are listed for each population. The red line indicates the cutoff of significance (adjusted p-value<0.05). The number of genes in each pathway is shown beside the pathway name. **Panel B. Heatmap of patients with complicated sarcoidosis and healthy controls.** Red represents increased gene expression; Blue represents down-regulation. “++”: patients with complicated sarcoidosis; “−”: healthy controls. **Panel C. Principal component analysis on expression values of the 20-gene signature**. X-axis: principal component 1 with eigenvalue; Y-axis: principal component 2 with eigenvalue. Left panel: patients with complicated sarcoidosis and healthy controls; middle panel: patients with complicated sarcoidosis, uncomplicated sarcoidosis and healthy controls; and right panel: patients with complicated sarcoidosis and uncomplicated sarcoidosis. HC: healthy controls; US: patients with uncomplicated sarcoidosis; and CS: patients with complicated sarcoidosis. **Panel D. Comparison between the 20-gene signature and the TCR/JS/CCR signaling pathway gene signature.** The distribution of prediction accuracy is based on 1,000 times of five-fold cross-validation. The dashed lines indicate the average classification accuracy for the 20-gene signature or the TCR/JS/CCR signaling pathway gene signature. Left panel: all sarcoidosis patients versus healthy controls; and right panel: patients with complicated sarcoidosis versus patients with uncomplicated sarcoidosis.

All cases with diagnoses of cardiac, neurologic, or severe pulmonary sarcoidosis (FVC<50%) comprised the cohort labeled as ‘complicated sarcoidosis’. At the specified significance level (fold-change >1.4, q-value <0.05), 316 genes were differentially expressed between all sarcoidosis cases and healthy controls in the combined samples (pooled AAs and EAs). For individual populations, 118 genes were differentially-expressed between all AA cases and controls, whereas 861 genes were differentially expressed between all EA cases and controls. In contrast, 1124 genes were differentially expressed between complicated sarcoidosis cases and healthy controls in the combined samples. For individual population, 730 and 980 genes were differentially expressed between AA and EA cases with complicated sarcoidosis and healthy controls, respectively with the TCR signaling pathway significantly enriched among complicated sarcoidosis-associated genes in both populations (adjusted P<0.05) (Figure 1A).

### Identifying a Gene Signature for Complicated Sarcoidosis

To identify a universal gene signature for complicated sarcoidosis in both AA and EA populations, an initial analysis set comprised of 1233 genes differentially expressed between AA or EA complicated sarcoidosis cases vs. healthy controls was utilized for the SVM algorithm. Figure S1 depicts the distribution of the prediction accuracy for gene signatures with the number of genes during recursive feature selection (see Supplementary Text S1 for details). A 20-gene signature (Table 3) was chosen as the most parsimonious signature with the peak prediction accuracy (Figure S1) and accurately distinguished patients with complicated sarcoidosis from healthy controls (Figures 1B and 1C), or from uncomplicated sarcoidosis (Figure 1C). Two genes within the unbiased 20-gene signature, *HBEGF* (heparin-binding EGF-like growth factor) and *SAP30* (Sin3A-associated protein, 30kDa), were strongly up-regulated in complicated sarcoidosis whereas the remaining 18 signature genes were down-regulated in complicated sarcoidosis (Figure S1). The non-targeted 20-gene signature distinguished all sarcoidosis patients from healthy controls with an accuracy of 86.0% (sensitivity = 88.2% and specificity = 83.3%) in the combined samples (pooled AAs and EAs) (Figure 1D). The discriminative accuracy became 88.2% and 94.2% in separating sarcoidosis cases from healthy controls in AA and EA, respectively (Figure S2). When distinguishing complicated sarcoidosis cases from uncomplicated sarcoidosis cases, the accuracy was 81.4% (sensitivity = 87.0% and specificity = 74.2%) in the combined samples (Figure 1D) but was reduced to 83.7% and 64.5% in separating complicated sarcoidosis cases from uncomplicated sarcoidosis cases in AA and EA, respectively (Figure S2).

**Table 3:** The unbiased 20-gene signature for complicated sarcoidosis.

Gene symbol	Gene title	Weight
*FITM2*	fat storage-inducing transmembrane protein 2	0.04872
*HBEGF*	heparin-binding EGF-like growth factor	0.04791
*TSHZ2*	teashirt zinc finger homeobox 2	0.04648
*MEI1*	meiosis inhibitor 1	0.04218
*LOC100287290*	cytokine receptor CRL2	0.03851
*ZNF540*	zinc finger protein 540	0.03776
*SAP30*	Sin3A-associated protein, 30kDa	0.02935
*ZNF614*	zinc finger protein 614	0.02715
*KIAA1147*	KIAA1147	0.02585
*LOC100132356*	hypothetical protein LOC100132356	0.02561
*CX3CR1*	chemokine (C-X3-C motif) receptor 1	0.02547
*RBM12B*	RNA binding motif protein 12B	0.02286
*FKBP1A*	FK506 binding protein 1A, 12kDa	0.02157
*SERTAD1*	SERTA domain containing 1	0.02119
*APOBEC3D*	apolipoprotein B mRNA editing enzyme, catalytic polypeptide-like 3D	0.02106
*KLRB1*	killer cell lectin-like receptor subfamily B, member 1	0.01979
*CRIP1*	cysteine-rich protein 1 (intestinal)	0.01889
*NOG*	noggin	0.01724
*SESN3*	sestrin 3	0.01701
*ZNF671*	zinc finger protein 671	0.01657

Here, the weight of each gene represents the frequency of the gene being selected during the last round of RFE procedure.

### Evaluation of a Sarcoidosis-related TCR/JS/CCR Signaling Pathway Gene Signature

As the T cell receptor pathway (TCR), the JAK STAT signaling pathway (JS) and the cytokine-cytokine receptor signaling pathway (CCR) have all been implicated in sarcoidosis [6], [26], a 31 gene signature comprised of TCR/JS/CCR signaling pathway genes implicated associated with sarcoidosis was assessed as a potential molecular biomarker in identifying cases or risk for complicated sarcoidosis (Table 4) (see Supplementary Text S1 for details). Overall, this TCR/JS/CCR signaling pathway signature differentiated sarcoidosis from healthy controls with a prediction accuracy of 82.2% (Figure 1D), but exhibited a substantially reduced prediction accuracy of <60% in distinguishing complicated sarcoidosis from uncomplicated sarcoidosis (Figure 1D). The discriminative accuracy of this TCR/JS/CCR signature was 83.2% in separating all AA sarcoidosis patients from healthy controls but only 69.7% for distinguishing AA complicated sarcoidosis cases from uncomplicated sarcoidosis. Similarly, in EA cases, the accuracy of the TCR/JS/CCR signature was 75.1% for distinguishing sarcoidosis patients from healthy controls, but only 37.5% in distinguishing EA patients with complicated sarcoidosis from uncomplicated EA sarcoidosis cases. Comparison of the prediction accuracy in both the TCR/JS/CCR and unbiased 20-gene signatures in combined EA and AA cases (Figure 1D, Figure S3) revealed the superior performance of the unbiased 20-gene sarcoidosis signature (P<10^−15^ by t-test). Finally, as sarcoidosis and IPF represent the most common interstitial lung diseases (ILDs) of unknown etiology, the capacity for the unbiased 20-gene and TCR/JS/CCR sarcoidosis gene signatures to distinguish sarcoidosis cases from IPF cases (GEO - GSE38958) was assessed. Each signature performed with comparable prediction accuracy in IPF and sarcoid with the 20-gene signature (77.2%) slightly superior to the TCR/JS/CCR signaling pathway signature (76.5%) in distinguishing sarcoidosis from IPF cases (Figure S4, P<10^−5^ by t-test).

**Table 4:** The 31 differentially-expressed TCR/JS/CRR signaling pathway genes in sarcoidosis.

		AA		EA	
Gene symbol	Gene title	Fold change	FDR (%)	Fold change	FDR (%)
*CD247*	CD247 molecule	0.63	0.0	0.62	0.2
*CD28*	CD28 molecule	0.60	0.3	0.55	0.5
*CD3D*	CD3d molecule, delta (CD3-TCR complex)	0.52	0.2	0.44	0.0
*CD3E*	CD3e molecule, epsilon (CD3-TCR complex)	0.67	1.0	0.49	0.2
*CD3G*	CD3g molecule, gamma (CD3-TCR complex)	0.52	0.2	0.45	0.0
*CD8A*	CD8a molecule	0.84	7.4	0.63	0.8
*CBLB*	Cas-Br-M (murine) ecotropic retroviral transforming sequence b	0.68	0.5	0.65	0.2
*GRAP2*	GRB2-related adaptor protein 2	0.84	7.4	0.71	1.2
*ITK*	IL2-inducible T-cell kinase	0.52	0.2	0.42	0.0
*NCK1*	NCK adaptor protein 1	0.88	11.3	0.71	0.2
*RASGRP1*	RAS guanyl releasing protein 1 (calcium and DAG-regulated)	0.51	0.0	0.48	0.0
*DLG1*	discs, large homolog 1 (Drosophila)	0.73	1.5	0.69	0.8
*ICOS*	inducible T-cell co-stimulator	0.59	0.2	0.61	1.7
*IFNG*	interferon, gamma	0.56	0.0	0.74	4.7
*IL7R*	interleukin 7 receptor	0.69	3.4	0.51	0.5
*JUN*	jun oncogene	0.67	4.3	2.17	23.7
*LCK*	lymphocyte-specific protein tyrosine kinase	0.70	0.5	0.61	0.2
*MAPK9*	mitogen-activated protein kinase 9	0.78	3.4	0.71	0.5
*MALT1*	mucosa associated lymphoid tissue lymphoma translocation gene 1	0.66	0.3	0.69	0.5
*NFATC2*	nuclear factor of activated T-cells, cytoplasmic, calcineurin-dependent 2	0.59	0.0	0.57	0.0
*NFATC3*	nuclear factor of activated T-cells, cytoplasmic, calcineurin-dependent 3	0.66	0.0	0.71	0.2
*PIK3CA*	phosphoinositide-3-kinase, catalytic, alpha polypeptide	0.76	2.3	0.69	0.5
*PLCG1*	phospholipase C, gamma 1	0.60	0.0	0.62	0.2
*AKT3*	v-akt murine thymoma viral oncogene homolog 3 (protein kinase B, gamma)	0.60	0.3	0.54	0.0
*ZAP70*	zeta-chain (TCR) associated protein kinase 70kDa	0.72	0.5	0.66	0.5
*CCND2*	cyclin D2	0.62	0.3	0.51	0.0
*IL2RA*	interleukin 2 receptor, alpha	0.61	0.3	0.78	17.2
*IL2RB*	interleukin 2 receptor, beta	0.66	0.3	0.64	1.7
*STAT4*	signal transducer and activator of transcription 4	0.61	0.3	0.52	0.0
*SPRED1*	sprouty-related, EVH1 domain containing 1	0.60	0.0	0.77	4.7
*SOCS4*	suppressor of cytokine signaling 4	0.71	0.3	0.77	0.8

EA: Caucasian Americans; AA: African Americans; FDR: false discovery rate.

### Validation on Independent Datasets

**Figure 2:**
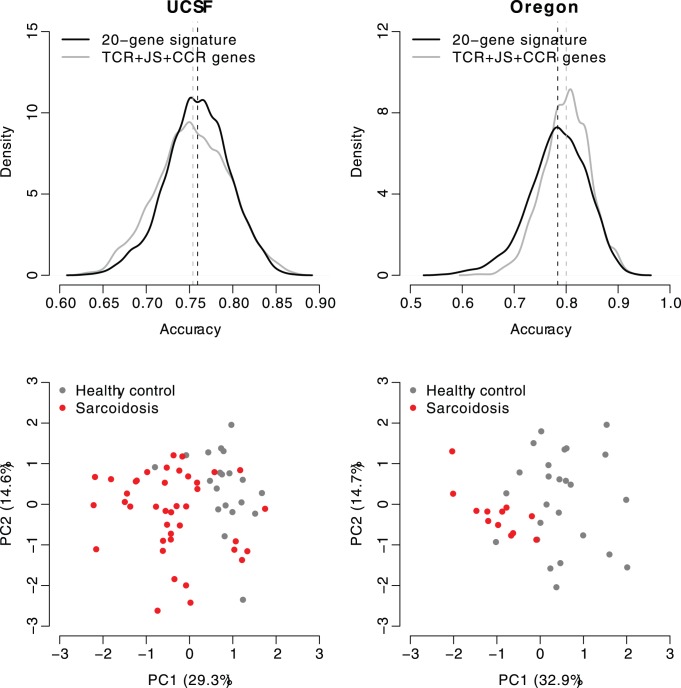
Validation in independent datasets. The upper panels show the comparison between the 20-gene signature and the TCR/JS/CCR signaling pathway gene signature. The distribution of prediction accuracy is based on 1,000 times of five-fold cross-validation. The dashed lines indicate the average classification accuracy for the 20-gene signature or the TCR/JS/CCR signaling pathway gene signature. The lower panels show the results of principal component analysis on expression values of the 20-gene signature. X-axis: principal component 1 with eigenvalue; Y-axis: principal component 2 with eigenvalue.

We evaluated the performance of our gene signatures in two different independent sarcoidosis blood gene expression datasets. One dataset (GEO - GSE19314) from University of California, San Francisco (UCSF) [27] and another one (GEO - GSE18781) is from Oregon Health Sciences University (Oregon) [28]. The discriminative power is very similar between the unbiased 20-gene and the TCR/JS/CCR signatures in the both datasets. The 20-gene signature classified sarcoidosis cases from healthy controls with accuracy of 75.9% and 78.3% for the USCF and Oregon datasets, respectively, while the discriminative accuracy became 75.4% and 80.0% when the TCR/JS/CCR signature was applied for the USCF and Oregon datasets, respectively (Figure 2). Again, principal component analysis indicates that patients with sarcoidosis can be well distinguished from healthy controls in the two independent datasets, just based on the expression of our unbiased 20-gene signature (Figure 2).

### Use of Genetic Variants to Validate Sarcoidosis Gene Signatures

A genome-wide association study (GWAS) (Affymetrix 6.0 SNP array) involving 407 sarcoidosis cases including 212 AAs (including 68 complicated cases) and 195 EAs (including 46 complicated cases) was performed and allele frequencies of ∼1,300 common SNPs residing in unbiased sarcoidosis signature genes analyzed in sarcoidosis cases and healthy controls (see Supplementary Text S1 for details). At the nominal P-value <0.01, 30 SNPs from 6 unbiased 20-gene signature genes were found to be significantly associated with sarcoidosis (Table 5), including 4 genes which overlapped between the AA and EA samples (*NOG* [noggin], *RMB12B* [RNA binding motif protein 12B], *SESN3* [sestrin 3], *TSHZ2* [teashirt zinc finger homeobox 2]). The most highly significant signature gene SNP in AAs was rs629508 (P = 1.7×10^−3^) in *SESN3*, whereas in EA cases, the most significant SNP was rs2618134 (P = 4.7×10^−5^) in *RBM12B*. Interestingly, several SNPs were also significantly associated with complicated sarcoidosis, including rs629508 (P = 5.4×10^−5^) and rs1294689 (P = 3.6×10^−5^) in the AA samples and rs10485815 (P = 2.8×10^−5^) in the EA samples (Table 5). In comparison, from ∼3,800 common SNPs residing in TCR/JS/CCR signature genes, 37 SNPs were associated with sarcoidosis in AA samples, whereas 34 SNPs were significant in EA samples, respectively (Table S1). The most highly significant TCR-JS-CCR signature gene SNP in AAs was rs2131817 (P = 1.4×10^−5^) in *AKT3*, whereas in EA cases, the most significant SNP was rs7614488 (P = 7.8×10^−7^) in *CBLB*. Several TCR/JS/CCR signature gene SNPs, rs2953040 and rs6791765 in *CBLB* (Cas-Br-M, murine, ecotropic retroviral transforming sequence b) and rs2131817 in *AKT3* were significantly associated with sarcoidosis in both EA and AA sarcoidosis cases (P<0.01) (Table S1).

**Table 5:** SNPs significantly associated with sarcoidosis within the unbiased 20 signature genes (P<0.01).

Population	SNP chromosome	dbSNP RS ID	Gene symbol	Gene relationship	Sarcoidosis vs healthy controls		Complicated sarcoidosis vs uncomplicated sarcoidosis	
					P	OR	P	OR
African Americans	11	rs629508	SESN3	intron	1.7E-03	1.645	5.4E-05	0.254
	17	rs7219027	NOG	downstream	4.3E-03	1.487		
	20	rs1294689	FKBP1A	intron	4.8E-03	1.536	3.6E-05	2.710
	11	rs12280779	SESN3	upstream	5.5E-03	1.555		
	20	rs201812	TSHZ2	intron	7.5E-03	1.438		
	8	rs16914980	RBM12B	downstream	7.8E-03	0.475		
	8	rs491546	RBM12B	downstream	8.9E-03	0.529		
	8	rs7821394	RBM12B	downstream	9.7E-03	0.728		
European Americans	8	rs2618134	RBM12B	downstream	4.7E-05	2.183		
	8	rs6993453	RBM12B	downstream	3.1E-04	1.819		
	20	rs1293381	TSHZ2	intron	3.8E-04	0.614		
	8	rs2595613	RBM12B	downstream	4.3E-04	2.357		
	8	rs12544183	RBM12B	downstream	5.3E-04	1.916		
	17	rs1914986	NOG	downstream	9.6E-04	1.946		
	8	rs279959	RBM12B	downstream	1.2E-03	1.621		
	3	rs4676483	CX3CR1	downstream	1.7E-03	1.671		
	8	rs10808648	RBM12B	downstream	2.0E-03	1.540		
	20	rs1326861	TSHZ2	downstream	2.1E-03	1.469		
	11	rs11021203	SESN3	upstream	4.0E-03	0.570		
	11	rs16922328	SESN3	upstream	5.5E-03	1.599		
	20	rs6068555	TSHZ2	intron	6.1E-03	0.710		
	8	rs549043	RBM12B	downstream	6.2E-03	1.805		
	8	rs566469	RBM12B	downstream	6.4E-03	1.718		
	20	rs6097326	TSHZ2	intron	6.4E-03	0.569		
	20	rs6068566	TSHZ2	downstream	6.6E-03	0.706		
	3	rs6773586	CX3CR1	upstream	8.3E-03	0.560		
	8	rs278586	RBM12B	downstream	9.0E-03	1.821		
	8	rs7829923	RBM12B	downstream	9.2E-03	1.434		
	17	rs17820808	NOG	downstream	9.3E-03	0.471		
	20	rs10485815	TSHZ2	intron	9.8E-03	1.653	2.8E-05	3.535

GWAS results between complicated and uncomplicated sarcoidosis were listed only for the SNPs with P<0.01. OR: odds ratio.

### PubMatrix Evaluation

The medical informatic tool PubMatrix (http://pubmatrix.grc.nia.nih.gov) tool was next used to evaluate the relevance of sarcoidosis signature genes in the published biomedical literatures (PubMed). Each signature gene was searched against a series of terms related to lung fibrosis or sarcoidosis including: “sarcoidosis”, “tuberculosis”, “granulomatous disease”, “hypersensitivity pneumonitis”, and “pulmonary fibrosis”. The majority of 20-gene signature genes were highly novel to these terms (Table 6) with only 2/20 genes having any PubMed citations linked to these terms (*HBEGF*, *LOC100132356*). Of the 31 TCR/JS/CCR-gene signature genes, 8/31 genes were cited in the sarcoidosis literature with *CD28*, *IFNG*, *IL7R*, *AKT3*, *IL2RA*, *IL2RB*, and *STAT4* demonstrating a robust relationship with these terms (Table S2).

**Table 6:** PubMatrix search results for the 20-gene signature against sarcoidosis-related search terms.

Gene	Sarcoidosis	Tuberculosis	Granulomatous disease	Hypersensitivity pneumonitis	Pulmonary fibrosis
*FITM2*	0	0	0	0	0
*HBEGF*	1	0	0	0	4
*TSHZ2*	0	0	0	0	0
*MEI1*	0	0	0	0	0
*LOC100287290*	0	0	0	0	0
*ZNF540*	0	0	0	0	0
*SAP30*	0	0	0	0	0
*ZNF614*	0	0	0	0	0
*KIAA1147*	0	0	0	0	0
*LOC100132356*	2	114	5	3	3
*CX3CR1*	0	0	0	0	0
*RBM12B*	0	0	0	0	0
*FKBP1A*	0	0	0	0	0
*SERTAD1*	0	0	0	0	0
*APOBEC3D*	0	0	0	0	0
*KLRB1*	0	0	0	0	0
*CRIP1*	0	0	0	0	0
*NOG*	0	0	0	0	0
*SESN3*	0	0	0	0	0
*ZNF671*	0	0	0	0	0

Each number in the table represents the count of literatures containing the corresponding gene name and search term.

## Discussion

**Figure 3:**
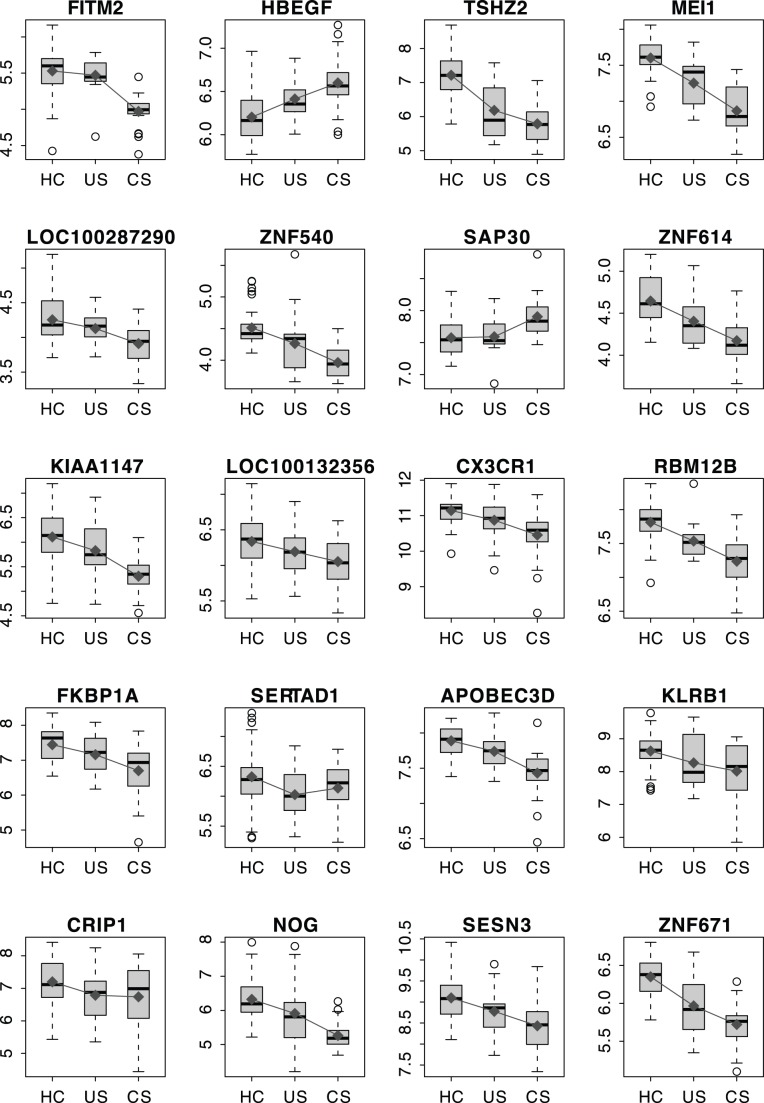
Boxplot of expression of the 20 signature genes. The dark grey points and lines indicate the geometric mean of expression in each category. HC: healthy controls; US: patients with uncomplicated sarcoidosis; and CS: patients with complicated sarcoidosis. Y-axis: log_2_-transformed expression values.

The major aim of this work was to identify potential universal and racially-specific gene signatures to serve as novel biomarkers for the presence of sarcoidosis as well as for the presence and/or susceptibility of the development of complicated sarcoidosis. Leveraging whole genome expression profiles in a cohort of sarcoidosis patients, an unbiased gene signature comprised of 20 autosomal genes was identified which distinguished sarcoidosis cases from healthy individuals and, importantly, differentiated patients with complicated sarcoidosis from patients with uncomplicated sarcoidosis. The 20-gene signature exhibited equivalent prediction accuracy to other sarcoidosis signatures containing a greater number of genes (such as 39-gene and 78-gene sarcoidosis signatures) with each signature superior in accuracy to signatures with fewer genes (e.g., the 10 gene signature) (Figure S1). The expression levels of the majority of these 20 signature genes showed a pattern of an additive model between uncomplicated and complicated sarcoidosis (Figure 3), i.e., when the signature gene is up-regulated, patients with complicated sarcoidosis exhibited higher expression levels than patients with uncomplicated sarcoidosis. In the sarcoidosis signature, 19 of 20 genes performed unidirectionally (up-regulation or down-regulation) in both complicated and uncomplicated sarcoidosis. Therefore, the 20-gene signature appears to not only capture differences between complicated sarcoidosis and healthy controls, but potentially conveys information regarding differences between sarcoidosis cases (both complicated and uncomplicated) and healthy controls.

Gene products encoded by TCR/JS/CCR signaling pathway genes have been implicated in sarcoidosis pathogenesis [6], [26] and these signature genes were enriched among the differential genes between EA and AA cases with complicated sarcoidosis cases and healthy controls. The utility of a TCR/JS/CCR signaling pathway gene signature in classifying sarcoidosis cases was compared to the unbiased 20-gene signature. Both signatures performed with high level prediction accuracy (>80%) in distinguishing cases with sarcoidosis from healthy controls. In contrast, the prediction accuracy of the 20-gene signature was much superior to the TCR/JS/CCR signaling pathway gene signature in classifying combined AA and EA patients with complicated and uncomplicated sarcoidosis (81.4% vs. 58.8%, P<10^−15^, t-test). We speculate that the unbiased nature of the 20-gene signature allows better capture of the characteristics of complicated sarcoidosis compared to the more restrictive TCR/JS/CCR signaling pathway signature genes. The potential role of TCR/JS/CCR signaling pathways genes in the development of sarcoidosis was confirmed by the capacity of this signature to successfully differentiate the majority of sarcoidosis and healthy controls. However, we speculate that either sarcoidosis disease progression or the development of complicated sarcoidosis likely requires the participation of genes and pathways extending beyond the TCR/JS/CCR pathway. These findings underscore the complex pathobiology of this disorder and implicate the necessity of global and unbiased approaches.

We further evaluated the classification accuracy of the 20-gene sarcoid signature separately in EA and AA samples and found the 20-gene signature to demonstrate >85% accuracy for classifying either EA or AA sarcoidosis cases (complicated and uncomplicated) from healthy controls. In contrast, the 20-gene sarcoidosis signature differentiated complicated sarcoidosis and uncomplicated sarcoidosis cases with an accuracy >80% in AA cases, but only ∼60% in EA cases, potentially the relative smaller complicated EA sample size or a bias for AA expression dysregulation driven by greater genetic variation, an issue which requires further examination. Both the 20-gene signature and TCR/JS/CCR-gene signature successfully discriminated sarcoidosis cases from IPF patients with similar prediction accuracies reflecting the differences in immunopathogenesis, clinical course, prognosis, and response to steroid treatment [29] in these two fibrotic lung disorders. This finding may infer additional clinical utility of the signature as a diagnostic biomarker for sarcoidosis.

As evidenced by the paucity of PubMed citations (PubMatrix results), the 20-gene signature is comprised of highly novel candidate genes in sarcoidosis susceptibility and severity of disease. As a complementary method to validate our findings [30]–[35], we examined the allele frequencies of both unbiased 20-gene sarcoidosis signature single nucleotide polymorphisms (SNPs) as well as TCR/JS/CCR signaling pathway signature gene SNPs in sarcoidosis cases and healthy controls embedded within a GWAS dataset constructed by genome-wide assessment of genetic variants in over 400 EA and AAs with sarcoidosis. As genetic variants, such as SNPs and copy number variants (CNVs), contribute significantly to variations in gene expression, SNPs were annotated to the genomic regions of these signature genes (based on the Affymetrix annotation) and, therefore, potentially contribute to gene expression variation by acting as *cis*-eQTLs. From ∼1,300 SNPs in our 20 signature genes, we identified 30 SNPs (corresponding to 6 signature genes) which were significantly associated with sarcoidosis in either EA or AA samples, suggesting a potential role of these cis-acting SNPs in regulating the expression of sarcoidosis signature genes. Similarly, from ∼3,800 SNPs in TCR/JS/CCR signature genes, relationships between SNPs and sarcoidosis were observed. While these findings serve to validate the potential importance and relevance of signature genes, a direct association between these SNPs and expression is necessary to validate these relationships. Our results suggest that genetic variants via *cis*-acting eQTLs may contribute to the variation in expression of sarcoidosis signature genes. We further recognize that additional factors, such as *trans*-acting eQTLs, environmental factors, or epigenetic pathways, may contribute substantially to signature gene expression variation. Further investigations involving genome-wide genotypic data (e.g., for mapping *trans*-acting eQTLs) and expression data on the same samples could potentially provide greater insights into the contribution of genetics to the identified gene signature.

Quantitative abnormalities in T cells have been described in the peripheral blood of patients with sarcoidosis [36] with significant lymphopenia, involving CD4, CD8, and CD19 positive cells, common in sarcoidosis patients and correlating with disease severity [37]. Individual signatures genes may not only have a role in the pathophysiology of sarcoidosis but could be potentially approached as novel therapeutic targets for the disease. For example, *HBEGF*, a member of the EGF family of growth factors, is a potent mitogen and chemoattractant for many cell types including fibroblasts, smooth muscle cells and epithelial cells [38]–[41]. A substantial body of evidence suggests that *HBGEF* plays a role in wound healing and response to injury [42]–[45] leading to speculation that *HBEGF* may represent a target involved in the pathobiology of chronic lung sarcoidosis and a novel therapeutic target, an observation supported by the PubMatrix search results.

Among our 20-gene signature, *LOC100132356* was most cited in PubMed literatures, though it only codes a hypothetical protein. This gene was linked to the terms such as sarcoidosis, tuberculosis, granulomatous disease, hypersensitivity pneumonitis, and pulmonary fibrosis. However, the detailed function of this gene is still unclear.

Recently, lung gene expression profiles were compared between patients with self-limiting sarcoidosis and those with progressive restrictive fibrotic disease [46] with a greater number of down-regulated genes versus up-regulated genes identified in patients with progressive pulmonary sarcoidosis. These findings are highly consistent with the expression profile of our signature genes in patients with complicated sarcoidosis. Interestingly, we failed to identify any overlap between sarcoidosis signature genes and the differentially expressed genes produced by comparison of self-limited and progressive lung sarcoidosis. The lack of overlap may reflect greater severity of disease in our cohort with cardiac and neurologic sarcoidosis in addition to cases with severe lung disease. In addition, our studies did not involve lung tissue expression but rather analysis of PBMCs and therefore tissue-specific expression may also contribute to this lack of overlap.

Furthermore, our sarcoidosis gene signatures performed well in two independent validation cohorts (UCSF and Oregon) [27], [28]. We should point out two challenges in our validation. Firstly, our microarray platform (Affymetrix Human Exon 1.0 ST Array) was different from that used for the validation cohorts (Affymetrix Human Genome U133 Plus 2.0 Array). Secondly, our study focused on gene expression in PBMCs while whole blood expression profiles were analyzed for the USCF cohort [27].

In summary, despite significant limitations including a relatively small size of the EA complicated cases in the analysis set, an unbiased 20-gene molecular gene signature was identified as a potential novel molecular biomarker in the diagnosis of sarcoidosis as well for the presence of complicated sarcoidosis with substantial accuracy in both EA and AA sarcoidosis cases. With validation in a replicate sarcoidosis cohort and testing against other granulomatous disorders like Wegener’s disease, hypersensitivity pneumonitis, and tuberculosis, this sarcoidosis gene signature may represent a novel universal gene signature for complicated sarcoidosis and serve as a springboard to individualized therapies in this enigmatic disorder.

## Materials and Methods

### Subjects and PBMC Samples

The study was approved by the Institutional Review Board (IRB) of the University of Illinois at Chicago (UIC) with written informed consent obtained from all subjects. The UIC’s IRB committee members (Chairs) include: Indru Punwani, D.D.S., Susan Labott, Ph.D., Paul Heckerling, M.D., and Kathryn Rugen, Ph.D. The DNA samples provided by the Johns Hopkins University investigators, and their use in this study, were approved by the IRB of the Johns Hopkins University. PBMC samples were collected from subjects with sarcoidosis (n = 39) and healthy controls (n = 35) (Table 1). The diagnosis of sarcoidosis was based on established joint international criteria [47]. Subjects with other concurrent systemic inflammatory diseases were excluded. A total of 29 African descent American (AA) and 10 European descent American (EA) patients with sarcoidosis were included in the overall sarcoidosis cohort with 18 AA and 4 EA patients diagnosed with complicated sarcoidosis defined as cardiac sarcoidosis (e.g., ventricular arrhythmias) [3], neurologic sarcoid (e.g., evidence of hyperdense MRI lesions) [4] or severe pulmonary sarcoidosis (FVC<50%). The detailed description of the therapy status of each patient has been listed in Table S3.

### RNA Microarray Hybridization

Total RNA was isolated from PBMCs using standard molecular biology protocols (n = 74) without DNA contamination or RNA degradation. Sample processing (e.g., cDNA generation, fragmentation, end labeling, hybridization to Affymetrix GeneChip Human Exon 1.0 ST arrays) was performed by the University of Chicago Functional Genomics Facility per manufacturer’s instructions.

### Identification of Genes Differentially Expressed in Sarcoidosis and Complicated Sarcoidosis

Human Exon 1.0 ST arrays were summarized using the Affymetrix Power Tools v.1.12.0 (http://www.affymetrix.com/) (see Supplementary Text S1 for details). The microarray data has been uploaded into NCBI GEO database (GEO accession number: GSE37912). Genes on chromosomes X and Y were removed to avoid the potential confounding factor of gender. SAM (Significance Analysis of Microarrays) [48], implemented in the *samr* library of the R Statistical Package [49], was used to compare log_2_-transformed gene expression levels between patients with complicated sarcoidosis and normal controls in the combined (AA and EA), EA, and AA samples, respectively. False discovery rate (FDR) was controlled using the q-value method [50]. Transcripts with a fold-change greater than 1.4 and q-value less than 0.05 were deemed differentially expressed. We searched for any enriched Kyoto Encyclopedia of Genes and Genomes (KEGG) [51] physiological pathways among the differential genes relative to the final analysis set using the NIH/DAVID [52], [53]. An adjusted P-value<0.05 after the Benjamini-Horchberg procedure [54] was used as the cutoff.

### Identification of Gene Signature for Classifying Sarcoidosis and Complicated Sarcoidosis

To identify gene signatures useful in the diagnosis and classification of sarcoidosis, a machine learning algorithm based on support vector machine (SVM) using a linear kernel, was applied in combination with recursive feature elimination (RFE) for generating a predictive model (see Supplementary Text S1 for details) [55]–[58]. The *e1071* library of the R Statistical Package [49] was used to conduct SVM and RFE. In each round of RFE, the SVM linear classifier was trained by the pooled samples from both AA and EA, including all the healthy controls and sarcoidosis patients. The gene signature that was comprised of the smallest number of genes with significant peak prediction accuracy was used in subsequent analyses. To test the performance of our gene signature, 1,000 times of five-fold cross-validation was conducted using SVM. In addition, the gene signature was also tested for classification accuracy in AA and EA samples, separately. We also used two independent sarcoidosis datasets using different microarray platforms [27], [28] to validate our gene signature.

## Supporting Information

Figure S1 **Distribution of the classification accuracy in each RFE step.** X-axis: the number of genes in each step; Y-axis: the classification accuracy from a five-fold cross-validation (repeated 1,000 times). The red line shows the average accuracy for each RFE step.
(PDF)

Figure S2 **Distribution of classification accuracies of the 20-gene signature.** X-axis: the classification accuracy from a five-fold cross-validation (repeated 1,000 times). The dashed lines indicate the average classification accuracy. (A) All sarcoidosis patients versus healthy controls in the AA samples; (B) Patients with complicated sarcoidosis versus patients with uncomplicated sarcoidosis in the AA samples; (C) All sarcoidosis patients versus healthy controls in the EA samples; and (D) Patients with complicated sarcoidosis versus patients with uncomplicated sarcoidosis in the EA samples.
(PDF)

Figure S3 **Comparison between the 20-gene signature and the TCR/JS/CCR signaling pathway gene signature in individual populations.** The distribution of accuracy is based on 1,000 times of five-fold cross-validation. The dashed lines indicate the average classification accuracy for the 20-gene signature or the TCR/JS/CCR signaling pathway gene signature. HC: healthy controls; US: patients with uncomplicated sarcoidosis; and CS: patients with complicated sarcoidosis.
(PDF)

Figure S4 **Capability of the the 20-gene signature and the TCR/JS/CCR signaling pathway gene signature in separating sarcoidosis patients from IPF patients.** The distribution of accuracy is based on 1,000 times of five-fold cross-validation. The dashed lines indicate the average classification accuracy for the 20-gene signature or the TCR/JS/CCR signaling pathway gene signature.
(PDF)

Table S1 **SNPs significantly associated with sarcoidosis within the 31 TCR/JS/CRR signature genes (P<0.01).**
(PDF)

Table S2 **PubMatrix search results for the TCR/JS/CCR signature genes against sarcoidosis-related search terms.**
(PDF)

Table S3 **Patient therapy description.**
(PDF)

Text S1 **Supplementary methods.**
(PDF)
