# Supplementary material for: Peripheral Blood Gene Expression as a Novel Genomic Biomarker in Complicated Sarcoidosis
Source: PLoS One. 2012 Sep 12;7(9):e44818. doi: 10.1371/journal.pone.0044818 (PMC3440319; doi:10.1371/journal.pone.0044818)
Supplement: Figure S1 — Distribution of the classification accuracy in each RFE step. X-axis: the number of genes in each step; Y-axis: the classification accuracy from a five-fold cross-validation (repeated 1,000 times). The red line shows the average accuracy for each RFE step. (PDF) [file pone.0044818.s001.pdf]

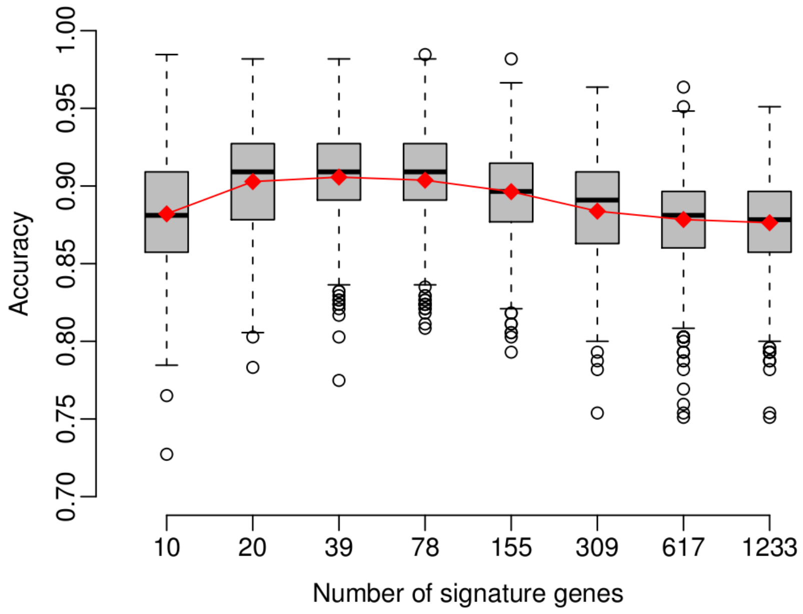

Figure S1. **Distribution of the classification accuracy in each RFE step.** X-axis: the number of genes in each step; Y-axis: the classification accuracy from a five-fold cross-validation (repeated 1,000 times). The red line shows the average accuracy for each RFE step.
