# Supplementary material for: Peripheral Blood Gene Expression as a Novel Genomic Biomarker in Complicated Sarcoidosis
Source: PLoS One. 2012 Sep 12;7(9):e44818. doi: 10.1371/journal.pone.0044818 (PMC3440319; doi:10.1371/journal.pone.0044818)
Supplement: Figure S2 — Distribution of classification accuracies of the 20-gene signature. X-axis: the classification accuracy from a five-fold cross-validation (repeated 1,000 times). The dashed lines indicate the average classification accuracy. (A) All sarcoidosis patients versus healthy controls in the AA samples; (B) Patients with complicated sarcoidosis versus patients with uncomplicated sarcoidosis in the AA samples; (C) All sarcoidosis patients versus healthy controls in the EA samples; and (D) Patients with complicated sarcoidosis versus patients with uncomplicated sarcoidosis in the EA samples. (PDF) [file pone.0044818.s002.pdf]

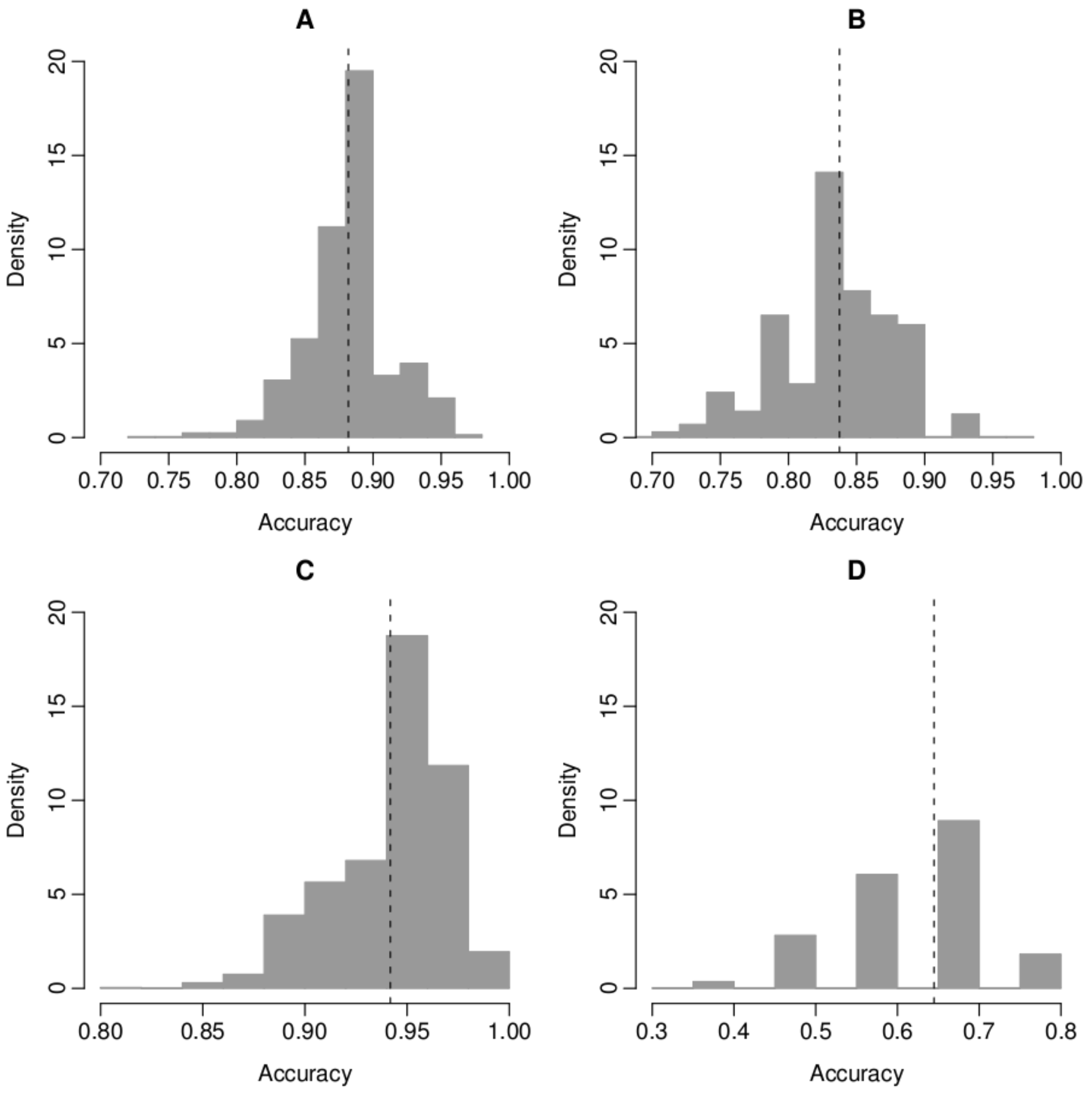

Figure S2. **Distribution of classification accuracies of the 20-gene signature.** X-axis: the classification accuracy from a five-fold cross-validation (repeated 1,000 times). The dashed lines indicate the average classification accuracy. (A) All sarcoidosis patients versus healthy controls in the AA samples; (B) Patients with complicated sarcoidosis versus patients with uncomplicated sarcoidosis in the AA samples; (C) All sarcoidosis patients versus healthy controls in the EA samples; and (D) Patients with complicated sarcoidosis versus patients with uncomplicated sarcoidosis in the EA samples.
