# Supplementary material for: Peripheral Blood Gene Expression as a Novel Genomic Biomarker in Complicated Sarcoidosis
Source: PLoS One. 2012 Sep 12;7(9):e44818. doi: 10.1371/journal.pone.0044818 (PMC3440319; doi:10.1371/journal.pone.0044818)
Supplement: Figure S3 — Comparison between the 20-gene signature and the TCR/JS/CCR signaling pathway gene signature in individual populations. The distribution of accuracy is based on 1,000 times of five-fold cross-validation. The dashed lines indicate the average classification accuracy for the 20-gene signature or the TCR/JS/CCR signaling pathway gene signature. HC: healthy controls; US: patients with uncomplicated sarcoidosis; and CS: patients with complicated sarcoidosis. (PDF) [file pone.0044818.s003.pdf]

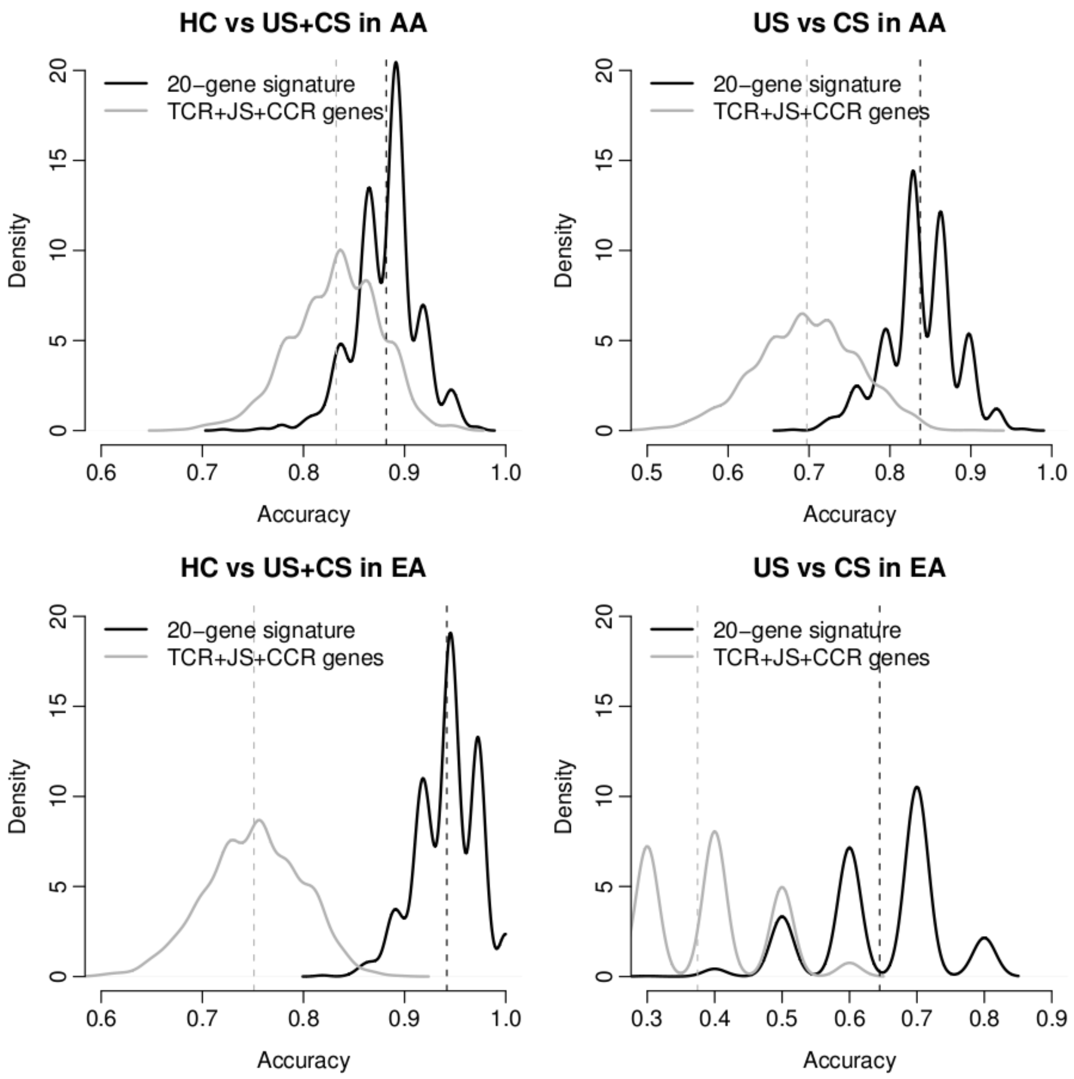

Figure S3. **Comparison between the 20-gene signature and the TCR/JS/CCR signaling pathway gene signature in individual populations.** The distribution of accuracy is based on 1,000 times of five-fold cross-validation. The dashed lines indicate the average classification accuracy for the 20-gene signature or the TCR/JS/CCR signaling pathway gene signature. HC: healthy controls; US: patients with uncomplicated sarcoidosis; and CS: patients with complicated sarcoidosis.
