# Supplementary material for: Peripheral Blood Gene Expression as a Novel Genomic Biomarker in Complicated Sarcoidosis
Source: PLoS One. 2012 Sep 12;7(9):e44818. doi: 10.1371/journal.pone.0044818 (PMC3440319; doi:10.1371/journal.pone.0044818)
Supplement: Figure S4 — Capability of the the 20-gene signature and the TCR/JS/CCR signaling pathway gene signature in separating sarcoidosis patients from IPF patients. The distribution of accuracy is based on 1,000 times of five-fold cross-validation. The dashed lines indicate the average classification accuracy for the 20-gene signature or the TCR/JS/CCR signaling pathway gene signature. (PDF) [file pone.0044818.s004.pdf]

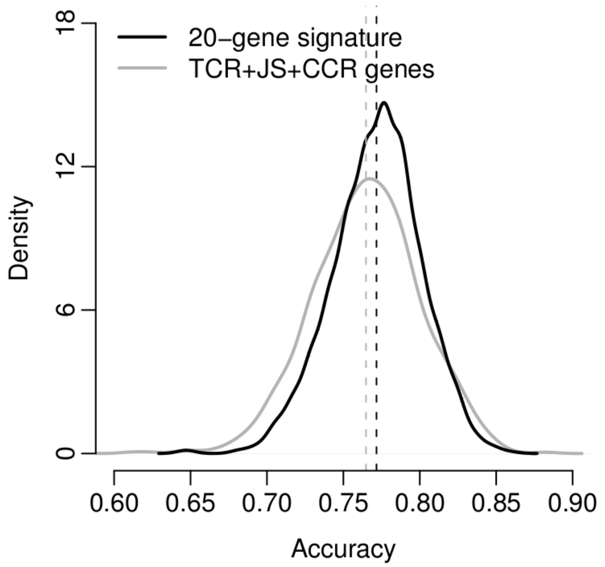

Figure S4. Capability of the the 20-gene signature and the TCR/JS/CCR signaling pathway gene signature in separating sarcoidosis patients from IPF patients. The distribution of accuracy is based on 1,000 times of five-fold cross-validation. The dashed lines indicate the average classification accuracy for the 20-gene signature or the TCR/JS/CCR signaling pathway gene signature.
