# Supplementary material for: Peripheral Blood Gene Expression as a Novel Genomic Biomarker in Complicated Sarcoidosis
Source: PLoS One. 2012 Sep 12;7(9):e44818. doi: 10.1371/journal.pone.0044818 (PMC3440319; doi:10.1371/journal.pone.0044818)
Supplement: Table S1 — SNPs significantly associated with sarcoidosis within the 31 TCR/JS/CRR signature genes (P<0.01). (PDF) [file pone.0044818.s005.pdf]

Table S1. SNPs significantly associated with sarcoidosis within the 31 TCR/JS/CRR signature genes (P < 0.01).

Population	SNP chromosome	dbSNP RS ID	Gene symbol	Gene relationship	Sarcoidosis vs healthy controls		Complicated sarcoidosis vs uncomplicated sarcoidosis	
					P	OR	P	OR
African Americans	1	rs2131817	AKT3	upstream	1.4E-05	2.061	3.8E-03	2.087
	3	rs2953040	CBLB	upstream	1.8E-05	0.584	3.8E-05	0.344
	10	rs2256867	IL2RA	intron	1.8E-04	1.823		
	5	rs11567719	IL7R	intron	3.1E-04	2.051		
	3	rs9855440	CBLB	upstream	3.4E-04	0.648		
	15	rs8032887	SPRED1	upstream	8.1E-04	0.329		
	1	rs1458023	AKT3	intron	8.7E-04	0.659		
	12	rs10492199	IFNG	downstream	8.8E-04	1.899		
	3	rs17202341	CBLB	intron	1.0E-03	0.461		
	3	rs6791765	CBLB	intron	1.8E-03	1.717		
	3	rs9831914	CBLB	upstream	2.9E-03	0.587		
	10	rs2245675	IL2RA	intron	3.3E-03	0.645		
	10	rs1887027	IL2RA	upstream	3.5E-03	1.453		
	22	rs11089825	IL2RB	upstream	4.4E-03	0.446		
	10	rs2476491	IL2RA	intron	5.3E-03	0.615		
	3	rs2676364	CBLB	upstream	5.6E-03	1.442		
	12	rs10784673	IFNG	downstream	5.9E-03	1.526		
	3	rs7620084	CBLB	upstream	7.3E-03	0.611		
	12	rs4332594	CCND2	downstream	8.1E-03	0.562		
	10	rs6602364	IL2RA	downstream	8.2E-03	0.721		
	1	rs2131817	AKT3	upstream	1.4E-05	2.061	3.8E-03	2.087
	3	rs2953040	CBLB	upstream	1.8E-05	0.584	3.8E-05	0.344
	5	rs11567719	IL7R	intron	3.1E-04	2.051		
	3	rs9855440	CBLB	upstream	3.4E-04	0.648		
	1	rs1458023	AKT3	intron	8.7E-04	0.659		
	12	rs10492199	IFNG	downstream	8.8E-04	1.899		
	3	rs17202341	CBLB	intron	1.0E-03	0.461		
	1	rs2716129	JUN	upstream	1.1E-03	0.667		
	3	rs6791765	CBLB	intron	1.8E-03	1.717		
	20	rs12106125	NFATC2	intron	2.7E-03	0.405		
	3	rs9831914	CBLB	upstream	2.9E-03	0.587		
	20	rs8119787	NFATC2	intron	3.5E-03	1.423		
	20	rs6102292	PLCG1	intron	3.6E-03	1.561		
	1	rs6684750	JUN	upstream	3.8E-03	1.505		
	5	rs163433	ITK	intron	3.9E-03	0.689		

	11	rs12788933	CD3E	upstream	4.9E-03	1.772		
	18	rs8094200	MALT1	downstream	5.4E-03	1.404		
	1	rs1773560	CD247	intron	5.5E-03	0.580		
	3	rs2676364	CBLB	upstream	5.6E-03	1.442		
	12	rs10784673	IFNG	downstream	5.9E-03	1.526		
	1	rs1723018	CD247	intron	6.4E-03	0.678		
	3	rs7620084	CBLB	upstream	7.3E-03	0.611		
	18	rs7231244	MALT1	upstream	9.0E-03	0.584		
	1	rs7556224	JUN	upstream	1.0E-02	0.730		
European Americans	3	rs7614488	CBLB	upstream	7.8E-07	2.495		
	3	rs2953040	CBLB	upstream	1.6E-04	1.615	3.7E-04	0.379
	3	rs4855606	CBLB	upstream	5.0E-04	0.344		
	3	rs16853082	CBLB	upstream	1.2E-03	0.514		
	3	rs11712635	CBLB	upstream	1.3E-03	1.830		
	3	rs2248981	CBLB	upstream	1.5E-03	0.664		
	3	rs767151	CBLB	upstream	1.7E-03	1.808		
	3	rs1520717	CBLB	upstream	1.7E-03	0.646		
	22	rs11089825	IL2RB	upstream	2.2E-03	0.591		
	3	rs6791765	CBLB	intron	2.5E-03	1.835		
	3	rs7641825	CBLB	intron	2.8E-03	1.585		
	15	rs17561445	SPRED1	upstream	2.8E-03	1.672	1.8E-03	0.288
	3	rs2049118	CBLB	upstream	3.4E-03	0.660		
	3	rs2655019	CBLB	upstream	3.4E-03	0.644		
	3	rs9863794	CBLB	upstream	3.5E-03	1.554		
	12	rs2870957	IFNG	downstream	3.9E-03	1.504		
	3	rs16852512	CBLB	upstream	4.7E-03	0.701		
	3	rs9826409	CBLB	upstream	5.3E-03	1.898		
	3	rs10433373	CBLB	downstream	6.5E-03	1.511		
	3	rs767150	CBLB	upstream	6.9E-03	1.696		
	3	rs10511245	CBLB	downstream	6.9E-03	1.506		
	3	rs9845005	CBLB	upstream	7.5E-03	1.461		
	3	rs10433370	CBLB	downstream	8.7E-03	1.489		
	1	rs2131817	AKT3	upstream	8.8E-03	0.633	2.3E-06	4.143
	3	rs9838585	CBLB	downstream	8.9E-03	1.513		
	3	rs9288812	CBLB	downstream	9.1E-03	1.484		
	3	rs4895039	CBLB	upstream	9.4E-03	0.694		
	3	rs7618427	CBLB	upstream	9.6E-03	0.605		
	3	rs6779049	CBLB	upstream	9.8E-03	0.690		
	3	rs7614488	CBLB	upstream	7.8E-07	2.495		
	3	rs2953040	CBLB	upstream	1.6E-04	1.615	3.7E-04	0.379
	3	rs4855606	CBLB	upstream	5.0E-04	0.344		

	3	rs16853082	CBLB	upstream	1.2E-03	0.514		
	3	rs11712635	CBLB	upstream	1.3E-03	1.830		
	3	rs2248981	CBLB	upstream	1.5E-03	0.664		
	3	rs767151	CBLB	upstream	1.7E-03	1.808		
	3	rs1520717	CBLB	upstream	1.7E-03	0.646		
	3	rs6791765	CBLB	intron	2.5E-03	1.835		
	3	rs7641825	CBLB	intron	2.8E-03	1.585		
	3	rs2049118	CBLB	upstream	3.4E-03	0.660		
	3	rs2655019	CBLB	upstream	3.4E-03	0.644		
	3	rs9863794	CBLB	upstream	3.5E-03	1.554		
	12	rs2870957	IFNG	downstream	3.9E-03	1.504		
	3	rs16852512	CBLB	upstream	4.7E-03	0.701		
	3	rs9826409	CBLB	upstream	5.3E-03	1.898		
	18	rs878875	MALT1	downstream	5.7E-03	1.431		
	3	rs10433373	CBLB	downstream	6.5E-03	1.511		
	2	rs11689629	CD28	downstream	6.5E-03	0.643		
	5	rs25781	ITK	intron	6.6E-03	0.698		
	3	rs767150	CBLB	upstream	6.9E-03	1.696		
	3	rs10511245	CBLB	downstream	6.9E-03	1.506		
	3	rs9845005	CBLB	upstream	7.5E-03	1.461		
	3	rs10433370	CBLB	downstream	8.7E-03	1.489		
	1	rs2131817	AKT3	upstream	8.8E-03	0.633	2.3E-06	4.143
	3	rs9838585	CBLB	downstream	8.9E-03	1.513		
	3	rs9288812	CBLB	downstream	9.1E-03	1.484		
	3	rs4895039	CBLB	upstream	9.4E-03	0.694		
	3	rs7618427	CBLB	upstream	9.6E-03	0.605		
	5	rs25780	ITK	intron	9.6E-03	0.711		
	3	rs3773853	DLG1	intron	9.7E-03	1.384		
	3	rs6779049	CBLB	upstream	9.8E-03	0.690		

GWAS results between complicated and uncomplicated sarcoidosis were listed only for the SNPs with P < 0.01. OR: odds ratio.
