# Supplementary material for: Peripheral Blood Gene Expression as a Novel Genomic Biomarker in Complicated Sarcoidosis
Source: PLoS One. 2012 Sep 12;7(9):e44818. doi: 10.1371/journal.pone.0044818 (PMC3440319; doi:10.1371/journal.pone.0044818)
Supplement: Table S2 — PubMatrix search results for the TCR/JS/CCR signature genes against sarcoidosis-related search terms. (PDF) [file pone.0044818.s006.pdf]

Table S2. PubMatrix search results for the TCR/JS/CCR signature genes against sarcoidosis-related search terms.

Gene	Sarcoidosis	Tuberculosis	Granulomatous disease	Hypersensitivity pneumonitis	Pulmonary fibrosis
CD247	0	0	0	0	0
CD28	9	38	20	6	8
CD3D	0	0	0	0	0
CD3E	0	0	0	0	0
CD3G	0	0	0	0	0
CD8A	0	0	0	0	0
CBLB	0	0	0	0	0
GRAP2	0	0	0	0	0
ITK	0	0	0	0	1
NCK1	0	0	0	0	0
RASGRP1	0	0	0	0	0
DLG1	0	0	0	0	0
ICOS	0	3	1	0	0
IFNG	188	3629	432	43	341
IL7R	2	8	4	0	0
JUN	0	1	3	1	28
LCK	1	3	0	0	1
MAPK9	0	1	0	0	0
MALT1	0	0	0	0	0
NFATC2	0	0	0	0	0
NFATC3	0	0	0	0	0
PIK3CA	0	0	0	0	1
PLCG1	0	2	3	0	3
AKT3	3	14	2	1	31
ZAP70	0	0	0	0	0
CCND2	0	0	0	0	0
IL2RA	3	27	5	1	2
IL2RB	10	43	8	3	4
STAT4	1	7	2	0	2
SPRED1	0	0	0	0	0
SOCS4	0	6	1	0	4

Each number in the table represents the count of literatures containing the corresponding gene name and search term.
