# Supplementary material for: Peripheral Blood Gene Expression as a Novel Genomic Biomarker in Complicated Sarcoidosis
Source: PLoS One. 2012 Sep 12;7(9):e44818. doi: 10.1371/journal.pone.0044818 (PMC3440319; doi:10.1371/journal.pone.0044818)
Supplement: Table S3 — Patient therapy description. (PDF) [file pone.0044818.s007.pdf]

Table S3. Patient therapy description.

ID	Complicated Sarcoidosis	Prednisone mg/day	MTX mg/week	Micophenolate mg/day	anti TNF therapy
Patient 1	No	10			
Patient 2	No	2.5			
Patient 3	No	40			
Patient 4	No		12		
Patient 5	No				
Patient 6	No	10			
Patient 7	No	5			
Patient 8	No				
Patient 9	No				
Patient 10	No			250	
Patient 11	No	15			
Patient 12	No				
Patient 13	No		12.5		
Patient 14	No				
Patient 15	No	40	12.5		
Patient 16	No				
Patient 17	No				
Patient 18	Yes	40			
Patient 19	Yes	10	10		
Patient 20	Yes	10			
Patient 21	Yes	10	10		
Patient 22	Yes		10		
Patient 23	Yes	10			
Patient 24	Yes	6.25		500	
Patient 25	Yes		7.5		
Patient 26	Yes			500	Yes
Patient 27	Yes		10		
Patient 28	Yes	5	15		
Patient 29	Yes		12		
Patient 30	Yes				
Patient 31	Yes		10		Yes
Patient 32	Yes	5			
Patient 33	Yes	15			
Patient 34	Yes	20	17.5		
Patient 35	Yes	5			
Patient 36	Yes	6		1000	
Patient 37	Yes				
Patient 38	Yes	10			Yes
Patient 39	Yes	20			Yes
