# Supplementary material for: Peripheral Blood Gene Expression as a Novel Genomic Biomarker in Complicated Sarcoidosis
Source: PLoS One. 2012 Sep 12;7(9):e44818. doi: 10.1371/journal.pone.0044818 (PMC3440319; doi:10.1371/journal.pone.0044818)
Supplement: Text S1 — Supplementary methods. (PDF) [file pone.0044818.s008.pdf]

Text S1. Supplementary methods

Microarray data preprocessing

Expression arrays were analyzed using the Affymetrix Power Tools v.1.12.0 (http://www.affymetrix.com/). The experimental probe masking workflow provided by the Affymetrix Power Tools was utilized to filter the probeset (exon-level) intensity files by removing probes that contain known SNPs in the dbSNP database (1) (v129). Overall, of the ~1.4 million probesets on the exon array, ~350,000 probesets were found to contain at least one probe with a SNP (~600,000 probes) (2). The resulting probe signal intensities were quartile normalized over all 74 samples. Probeset expression signals were summarized with the robust multi-array average (RMA) algorithm (3) and $\log_2$ transformed with a median polish. We then generated the expression signals of the ~22,000 transcript clusters (gene-level) with the core set (i.e., with RefSeq-supported annotations) (4) of exons by taking averages of all annotated probesets for each transcript cluster. Adjustment for possible batch effect was conducted by COMBAT (http://jlab.byu.edu//ComBat/) (5). We consider a transcript cluster to be reliably expressed in these samples if the Affymetrix implemented DABG (detection above ground) (6) p-value was less than 0.01 in at least 67% of the samples in each test group (healthy controls, patients with complicated sarcoidosis, patients with uncomplicated sarcoidosis) in each population, respectively. We further limited our analysis set to the genes with unique annotations (i.e., transcripts corresponding to unique genes) from the Affymetrix NetAffy website (https://www.affymetrix.com/analysis/netaffx/, accessed on Dec. 1, 2010). Totally, 11,412 and 11,592 transcript clusters in the AA and EA samples, respectively, met these criteria and were further analyzed.

Principal component analysis

Principal component analysis (PCA) was used to investigate the major trend in gene expression variation among patients. In this study, the expression values of genes in each patient were plotted in a multidimensional space. PCA identified a series of new orthogonal axes accounting for the greatest variation among patients. The analysis yielded the coordinate of each patient on each new axis, and the fraction of the total variation was accounted for by each axis.

Support vector machine

Support vector machine (SVM) is a machine learning technique based on statistical theory. The principle of SVM is to find a maximum margin hyperplane for classification. The instances are mapped to a higher dimensional space using the kernel function. Kernel function allows one to work in a higher dimensional space without computing all elements. SVM will then choose a maximum soft margin separating hyperplane in this higher dimensional space, which separates the training instances by their classes. The classification of a test sample will then be determined by a sign function which is defined by the parameters of the hyperplane. The instances closest to the hyperplane are called support vectors and are vital for training (7).

Predictive model to identify signature genes

To identify gene signatures useful in the diagnosis and classification of sarcoidosis, SVM using a linear kernel (7), was applied in combination with recursive feature elimination (RFE) for generating a predictive model. The decision function of a linear SVM is

$$f(\mathbf{x}) = sign\{(\mathbf{w} \bullet \mathbf{x}) + b\}$$
$$f(\mathbf{x}) = sign\{(\mathbf{w} \bullet \mathbf{x}) + b\}$$

where **x** is the gene expression vector of a sample, **w** is the vector of weights of the features, and b is a scalar offset (8-10). The RFE approach recursively reduces the number of genes used in the predictor function by removing those genes with lowest weights and re-fitting the SVM algorithm using the remaining genes. In the first step, all differentially expressed genes (at the above-mentioned significance level) between cases with complicated sarcoidosis and healthy AA or EA controls are ranked by SVM according to their weights. In each of the following steps of the RFE procedure, 50% of genes were eliminated from the predictor model until the gene number was less than 20. A five-fold cross-validation (repeated 1,000 times) of the predictive models based on differently sized subsets of genes, as selected by RFE, was performed. After the recursive feature selection steps on each subset, the frequency of selected features were counted at each level among all rounds of cross-validation experiments. The most frequently selected features were reported as signature genes (10).

Performance measurement of predictive model

The performance of the predictive model was evaluated by five-fold cross-validation accuracy, sensitivity, and specificity. These indices are determined thus:

$$Accuracy = (TP + TN)/(TP + TN + FP + FN)$$

$$Sensitivity = TP/(TP + FN)$$

$$Specificity = TN/(TN + FP)$$

where TP, TN, FP, and FN are the number of true positives, true negatives, false positives and false negatives, respectively.

Generation of a T cell receptor TCR/JAK-STAT/cytokine-cytokine receptor signaling pathway gene signature

T cell receptor (TCR) signaling pathway genes, as annotated by the KEGG (11), are comprised of the TCR and co-stimulatory molecules such as CD28 and *IL7R*, a gene highly expressed in both naïve and memory T cells and implicated in sarcoidosis susceptibility (12-14). Because the JAK-STAT (JS) and cytokine-cytokine receptor (CCR) signaling pathways are implicated in sarcoidosis pathogenesis, genes within these two pathways were also collected from KEGG (11). TCR/JS/CCR signaling pathway genes differentially expressed between EA or AA patients with complicated sarcoidosis and normal controls were estimated for their power to classify sarcoidosis cases and normal controls, as well as complicated and uncomplicated sarcoidosis in our combined (EA and AA), EA, and AA samples, separately. Using linear SVM, a five-fold cross-validation (repeated for 1,000 times) of the predictive models based on TCR/JS/CCR signaling pathway genes was performed. The means of the predictive accuracy of the TCR/JS/CCR signaling pathway genes were compared with those of a 20-gene signature by standard t test ($P < 0.05$ as the cutoff for significance).

Genotypic data on SNPs residing within sarcoidosis signature genes

We obtained genotypic data for signature gene SNPs via analysis of a sarcoidosis GWAS (genome-wide association study) with current SNP and gene annotations obtained from the Affymetrix NetAffy website (accessed on Dec. 1, 2010). The sarcoidosis GWAS dataset was comprised of 195 (46 complicated) EA cases and 212 (68 complicated) AA cases with SNPs genotyped using the Affymetrix 6.0 SNP Array. Briefly, the *SNPRMA* and *CRLMM* packages of the Bioconductor Project (15) were used to preprocess the scanned intensities and genotype

calling. Genotypic data were checked for genotyping rate and Hardy-Weinberg Equilibrium ($P < 10^{-6}$) and publicly available dbGaP (http://www.ncbi.nlm.nih.gov/gap) data for the GAIN Genome-wide Association Study of Schizophrenia (v3, October, 2010) utilized as healthy normal controls. Specifically, 1-1 matched dbGaP samples were selected based on general genetic background (i.e., according to the weighted distance between each case and controls from a principal component analysis on common SNPs with minor allele frequency (MAF) greater than 0.05 in normal individuals) and gender for each population. The allele frequencies of common SNPs (MAF>0.05) in signature genes and genes in candidate pathways were compared using PLINK (16) between patients and normal controls, as well as between complicated and uncomplicated sarcoidosis patients in each population, separately. Since this is a targeted analysis on a small number of signature and candidate genes, a cutoff of nominal p-value<0.01 was chosen to call significant relationships.

References

1. Sherry ST, Ward MH, Kholodov M, Baker J, Phan L, Smigielski EM, Sirotkin K. Dbsnp: The ncbi database of genetic variation. *Nucleic Acids Res* 2001;29:308-311.

2. Duan S, Zhang W, Bleibel WK, Cox NJ, Dolan ME. Snpinprobe_1.0: A database for filtering out probes in the affymetrix genechip(r) human exon 1.0 st array potentially affected by snps. *Bioinformation* 2008;2:469-470.

3. Irizarry RA, Hobbs B, Collin F, Beazer-Barclay YD, Antonellis KJ, Scherf U, Speed TP. Exploration, normalization, and summaries of high density oligonucleotide array probe level data. *Biostatistics* 2003;4:249-264.

4. Pruitt KD, Tatusova T, Maglott DR. Ncbi reference sequences (refseq): A curated non-redundant sequence database of genomes, transcripts and proteins. *Nucleic Acids Res* 2007;35:D61-65.

5. Johnson WE, Li C, Rabinovic A. Adjusting batch effects in microarray expression data using empirical bayes methods. *Biostatistics* 2007;8:118-127.

6. Affymetrix. Exon array background correction. *Affymetrix Whitepaper* 2005;http://media.affymetrix.com/support/technical/whitepapers/exon_background_correction_whitepaper.pdf.

7. Vapnik V. Statistical learning theory. New York: John Wiley & Sons; 1998.

8. Guyon I, Weston J, Barnhill S. Gene selection for cancer classification using support vector machines. *Machine Learning* 2002;46:389-422.

9. Thuerigen O, Schneeweiss A, Toedt G, Warnat P, Hahn M, Kramer H, Brors B, Rudlowski C, Benner A, Schuetz F, et al. Gene expression signature predicting pathologic complete response with gemcitabine, epirubicin, and docetaxel in primary breast cancer. *J Clin Oncol* 2006;24:1839-1845.

10. Zhang X, Lu X, Shi Q, Xu XQ, Leung HC, Harris LN, Iglehart JD, Miron A, Liu JS, Wong WH. Recursive svm feature selection and sample classification for mass-spectrometry and microarray data. *BMC Bioinformatics* 2006;7:197.

11. Kanehisa M, Goto S, Kawashima S, Okuno Y, Hattori M. The kegg resource for deciphering the genome. *Nucleic Acids Res* 2004;32:D277-280.

12. Heron M, Grutters JC, van Moorsel CH, Ruven HJ, Huizinga TW, van der Helm-van Mil AH, Claessen AM, van den Bosch JM. Variation in il7r predisposes to sarcoid inflammation. *Genes Immun* 2009;10:647-653.

13. Iannuzzi MC, Iyengar SK, Gray-McGuire C, Elston RC, Baughman RP, Donohue JF, Hirst K, Judson MA, Kavuru MS, Maliarik MJ, et al. Genome-wide search for sarcoidosis susceptibility genes in african americans. *Genes Immun* 2005;6:509-518.

14. Maver A, Medica I, Peterlin B. Search for sarcoidosis candidate genes by integration of data from genomic, transcriptomic and proteomic studies. *Med Sci Monit* 2009;15:SR22-28.

15. Gentleman RC, Carey VJ, Bates DM, Bolstad B, Dettling M, Dudoit S, Ellis B, Gautier L, Ge Y, Gentry J, et al. Bioconductor: Open software development for computational biology and bioinformatics. *Genome Biol* 2004;5:R80.

16. Purcell S, Neale B, Todd-Brown K, Thomas L, Ferreira MA, Bender D, Maller J, Sklar P, de Bakker PI, Daly MJ, et al. Plink: A tool set for whole-genome association and population-based linkage analyses. *Am J Hum Genet* 2007;81:559-575.
